# From spinal cord to hippocampus: links between bifurcation structure, ion channel expression, and firing patterns in a variety of neuron types

**DOI:** 10.1186/1471-2202-13-S1-P121

**Published:** 2012-07-16

**Authors:** Erin C McKiernan, Marco Arieli Herrera Valdez

**Affiliations:** 1 Institute of Interdisciplinary Research, University of Puerto Rico at Cayey, Cayey, PR, 00736, USA; 2 Department of Mathematics and Physics, University of Puerto Rico at Cayey, Cayey, PR, 00736, USA

## 

Neurons throughout the brain show remarkable diversity in their firing patterns, ranging from the generation of single action potentials to repetitive firing characterized by distinct latencies, firing frequencies, and spike shapes in response to current injection [3-5]. We present a minimal electrodiffusion-based model of membrane potential dynamics [1,2] to explore the diversity of firing patterns in a variety of neuron types, including spinal motor neurons and hippocampal granule cells. The minimal model includes voltage-gated sodium and potassium currents as well as a non-voltage-gated leak current. Our results demonstrate that small changes in the relative expression of potassium to sodium channels produce membranes with different underlying bifurcation structures. These bifurcation structures determine the way in which neurons transition from rest to spiking and, consequently, the types of firing patterns that can be observed. In addition, altering the activation kinetics of potassium channels produces additional changes in bifurcation structure, most notably, variations in the sequence of bifurcations that occur as the neuron moves from rest to spiking and back to rest again. Thus, we present not only a general biophysical model that can be used to explore firing pattern diversity in many different types of neurons, but demonstrate the links between bifurcation structure, relative ion channel expression, and firing patterns.
